# *catena*-Poly[[(*p*-toluene­sulfonato-κ*O*)silver(I)]-μ-1,3-bis­(pyridin-4-yl)propane-κ^2^ *N*:*N*′]

**DOI:** 10.1107/S1600536812015218

**Published:** 2012-04-18

**Authors:** Feng Ma, Dai-Bao Wei, Zhi-Min Cao

**Affiliations:** a Shandong Polytechnic University, School of Chemistry and Pharmaceutical Engineering, Jinan 250353, People’s Republic of China

## Abstract

In the title compound, [Ag(C_7_H_7_O_3_S)(C_13_H_14_N_2_)]_*n*_, the Ag^I^ ion is coordinated in a T-shape by two N atoms from two symmetry-related 1,3-bis­(pyridin-4-yl)propane ligands and one O atom from a *p*-toluene­sulfonate ligand, forming a one-dimensional zigzag chain along [001]. In the crystal, weak C—H⋯O hydrogen bonds and weak Ag⋯Ag inter­actions [3.2628 (5) Å] are observed.

## Related literature

For potential applications of compounds with metal-organic framework structures, see: Horike *et al.* (2008 ▶); Liu *et al.* (2010 ▶); Lu *et al.* (2006 ▶); Li *et al.* (1999 ▶). For coordination polymers of 1,3-bis­(pyridin-4-yl)propane (bpp), see: Carlucci *et al.* (2002 ▶). For mixed ligands of aromatic or aliphatic carboxyl­ates and bpp, see: Yang *et al.* (2009 ▶); Jin *et al.* (2009 ▶); Zhang *et al.* (2009 ▶); Luo *et al.* (2011 ▶). For silver(I) sulfonate complexes, see: Wu *et al.* (2011 ▶); Smith *et al.* (1998 ▶) For similar systems with Ag⋯Ag inter­actions, see: Li *et al.* (2005 ▶). For a similar synthetic procedure, see: Li *et al.* (2006 ▶).
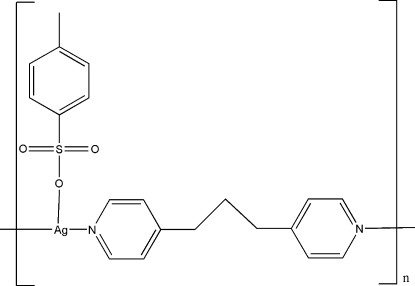


## Experimental

### 

#### Crystal data

[Ag(C_7_H_7_O_3_S)(C_13_H_14_N_2_)]
*M* *_r_* = 477.33
Monoclinic, 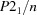

*a* = 10.8061 (3) Å
*b* = 9.9466 (3) Å
*c* = 18.4288 (5) Å
β = 98.230 (3)°
*V* = 1960.40 (10) Å^3^
*Z* = 4
Mo *K*α radiation
μ = 1.16 mm^−1^
*T* = 293 K
0.52 × 0.47 × 0.36 mm

#### Data collection

Agilent Xcalibur Eos Gemini diffractometer
Absorption correction: multi-scan (*CrysAlis PRO*; Agilent, 2010 ▶) *T*_min_ = 0.553, *T*_max_ = 0.659
11169 measured reflections
3450 independent reflections
2834 reflections with *I* > 2σ(*I*)
*R*_int_ = 0.030

#### Refinement

*R*[*F* ^2^ > 2σ(*F* ^2^)] = 0.030
*wR*(*F* ^2^) = 0.075
*S* = 0.94
3450 reflections
245 parameters
H-atom parameters constrained
Δρ_max_ = 0.63 e Å^−3^
Δρ_min_ = −0.55 e Å^−3^

### 

Data collection: *CrysAlis PRO* (Agilent, 2010 ▶); cell refinement: *CrysAlis PRO*; data reduction: *CrysAlis PRO*; program(s) used to solve structure: *SHELXS97* (Sheldrick, 2008 ▶); program(s) used to refine structure: *SHELXL97* (Sheldrick, 2008 ▶); molecular graphics: *SHELXTL* (Sheldrick, 2008 ▶) and *DIAMOND* (Brandenburg & Putz, 2004 ▶); software used to prepare material for publication: *SHELXL97*.

## Supplementary Material

Crystal structure: contains datablock(s) I, global. DOI: 10.1107/S1600536812015218/lh5431sup1.cif

Supplementary material file. DOI: 10.1107/S1600536812015218/lh5431Isup2.cdx

Structure factors: contains datablock(s) I. DOI: 10.1107/S1600536812015218/lh5431Isup3.hkl

Additional supplementary materials:  crystallographic information; 3D view; checkCIF report

## Figures and Tables

**Table 1:** Selected bond lengths (Å)

N1—Ag1^i^	2.155 (2)
N2—Ag1	2.162 (2)
O3—Ag1	2.645 (2)

Symmetry code: (i) 
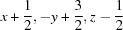
.

**Table 2:** Hydrogen-bond geometry (Å, °)

*D*—H⋯*A*	*D*—H	H⋯*A*	*D*⋯*A*	*D*—H⋯*A*
C14—H14*B*⋯O2^ii^	0.97	2.56	3.533 (4)	178
C12—H12⋯O1^i^	0.93	2.58	3.332 (4)	138
C8—H8⋯O3^iii^	0.93	2.46	3.305 (4)	151

Symmetry codes: (i) 
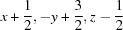
; (ii) 
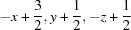
; (iii) 
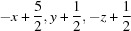
.

**Fig. 1.**
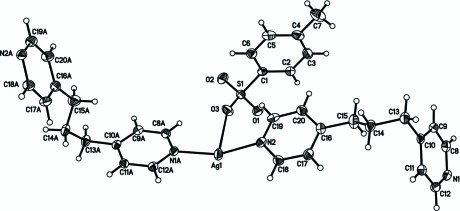
The molecular structure of (I), with 50% probability displacement ellipsoids for non-H atoms. Atoms labeled with capital "A" were generated through the symmetry operation (x - 1/2, -y + 3/2, z + 1/2).

**Fig. 2.**
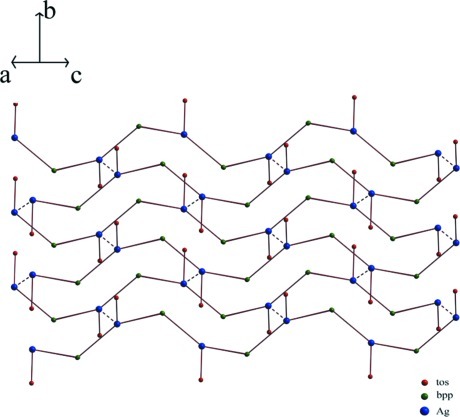
Part of the crystal structure. Tos and bpp ligands have been simplified by replacing molecules with their centroids (dummy atoms). Dashed lines indicate weak Ag···Ag interactions.

## supplementary crystallographic information

### Comment

The design and synthesis of metal-organic frameworks (MOFs) have attracted considerable attention in recent years,not only for their intriguing structural diversity, but also because of their potential applications in the area of catalysis (Horike *et al.*, 2008), ion exchange (Liu *et al.*, 2010), magnetism, photochemistry (Lu *et al.*, 2006), and porous materials (Li *et al.*, 1999).

The reactions of silver salts with 1,3-bis(pyridin-4-yl)propane (bpp) have already afforded intresting coordination polymers with distinct structural motifs (Carlucci *et al.*, 2002). Moreover, the combination of Ag cations with mixed ligands of aromatic (Yang *et al.*, 2009; Jin *et al.*, 2009; Zhang *et al.*, 2009) or aliphatic (Luo *et al.*, 2011) carboxylates and bpp can allow the formation of MOFs possessing fascinating architectures and novel topologies. In terms of silver(I) sulfonate complexes, many nitrogen-based secondary ligands such as pyrazine (Pyr) (Li *et al.*, 2005), hexamethylenetetramine (hmt) (Wu *et al.*, 2011), pyridine (py) (Smith *et al.*, 1998) and their analogues or derivatives were exploited as secondary ligands to synthesize new metal organic frameworks. Here we report a novel one-dimensional polymer [Ag(C_7_H_7_O_3_S)(C_13_H_14_N_2_)]_n_ assembled by mixed ligands of p-toluenesulfonate and bpp with silver nitrate.

The asymmetric unit of the title compound (I) and symmetry related atoms are shown in Fig. 1. The Ag^I^ cation is coordinated by two N atoms from two symmetry related bpp ligands one O atom from one p-toluenesulfonate ligand. The Ag—O and Ag—N bonds are comparable to those in the literature (Li *et al.*, 2005). As shown in Fig. 2, there are weak Ag···Ag interactions (3.2628 (5)Å) which are shorter than the sum van der Waals radii for Ag···Ag [3.40Å]. Compound (I) is a one-dimensional coordination polymer formed by bpp ligands and Ag cations, with tos ligands as the side chains in which bpp ligands exhibit the TG (Carlucci *et al.*, 2002) conformation and the N···N separation is 8.6746 (2)Å. The weak Ag ···Ag interactions bridge the undulating 1-D chains to form 2-D layers (Fig .2), which are further linked into a three-dimensional network via weak C—H···O hydrogen bonds.

### Experimental

A solution of bpp and tos in 8 ml methanol and 2 ml water was slowly added to a solution of AgNO_3_(0.1668 g) in 8 ml water. A white suspension formed immediately. An aqueous NH_3_ solution (25%) was dropped into the mixture to give a clear solution. The resultant colorless filtrate (pH=9.0) was allowed to evaporate slowly at room temperature. After one week, colorless block crystals were obtained in 52.87% yield (based on Ag). Elemental analysis for (I) (%): calculated: C 50.3, H 4.4, N 5.9, O 10.1, S 6.7. Found C49.94, H 4.452, N 5.969, O 10.123, S 7.013.

### Refinement

All H atoms were positioned geometrically and refined using a riding model with C—H = 0.93–0.98 Å and with *U*_iso_(H) = 1.2(1.5 for methyl groups) times *U*_eq_(C).

### Crystal data

**Table:**

[Ag(C_7_H_7_O_3_S)(C_13_H_14_N_2_)]	*F*(000) = 968
*M**_r_* = 477.33	*D*_x_ = 1.617 Mg m^−^^3^
Monoclinic, *P*2_1_/*n*	Mo *K*α radiation, λ = 0.7107 Å
Hall symbol: -P 2yn	Cell parameters from 4048 reflections
*a* = 10.8061 (3) Å	θ = 2.8–28.9°
*b* = 9.9466 (3) Å	µ = 1.16 mm^−^^1^
*c* = 18.4288 (5) Å	*T* = 293 K
β = 98.230 (3)°	Block, colorless
*V* = 1960.40 (10) Å^3^	0.52 × 0.47 × 0.36 mm
*Z* = 4	

### Data collection

**Table:**

Agilent Xcalibur Eos Gemini diffractometer	3450 independent reflections
Radiation source: Enhance (Mo) X-ray Source'	2834 reflections with *I* > 2σ(*I*)
Graphite monochromator	*R*_int_ = 0.030
φ and ω scans	θ_max_ = 25.0°, θ_min_ = 2.8°
Absorption correction: multi-scan (*CrysAlis PRO*; Agilent, 2010)	*h* = −12→12
*T*_min_ = 0.553, *T*_max_ = 0.659	*k* = −11→11
11169 measured reflections	*l* = −21→21

### Refinement

**Table:**

Refinement on *F*^2^	Secondary atom site location: difference Fourier map
Least-squares matrix: full	Hydrogen site location: inferred from neighbouring sites
*R*[*F*^2^ > 2σ(*F*^2^)] = 0.030	H-atom parameters constrained
*wR*(*F*^2^) = 0.075	*w* = 1/[σ^2^(*F*_o_^2^) + (0.032*P*)^2^ + 1.9473*P*] where *P* = (*F*_o_^2^ + 2*F*_c_^2^)/3
*S* = 0.94	(Δ/σ)_max_ = 0.001
3450 reflections	Δρ_max_ = 0.63 e Å^−^^3^
245 parameters	Δρ_min_ = −0.55 e Å^−^^3^
0 restraints	Extinction correction: *SHELXL*
Primary atom site location: structure-invariant direct methods	Extinction coefficient: 0.0029 (3)

### Special details

**Table:**

Geometry. All e.s.d.'s (except the e.s.d. in the dihedral angle between two l.s. planes) are estimated using the full covariance matrix. The cell e.s.d.'s are taken into account individually in the estimation of e.s.d.'s in distances, angles and torsion angles; correlations between e.s.d.'s in cell parameters are only used when they are defined by crystal symmetry. An approximate (isotropic) treatment of cell e.s.d.'s is used for estimating e.s.d.'s involving l.s. planes.
Refinement. Refinement of *F*^2^ against ALL reflections. The weighted *R*-factor *wR* and goodness of fit *S* are based on *F*^2^, conventional *R*-factors *R* are based on *F*, with *F* set to zero for negative *F*^2^. The threshold expression of *F*^2^ > σ(*F*^2^) is used only for calculating *R*-factors(gt) *etc*. and is not relevant to the choice of reflections for refinement. *R*-factors based on *F*^2^ are statistically about twice as large as those based on *F*, and *R*- factors based on ALL data will be even larger.

### Fractional atomic coordinates and isotropic or equivalent isotropic displacement parameters (Å^2^)

**Table:**

	*x*	*y*	*z*	*U*_iso_*/*U*_eq_	
C1	0.6485 (3)	0.3186 (3)	0.37306 (16)	0.0409 (7)	
C2	0.5674 (3)	0.2730 (3)	0.41963 (17)	0.0461 (8)	
H2	0.5350	0.3328	0.4508	0.055*	
C3	0.5348 (3)	0.1389 (4)	0.4196 (2)	0.0555 (9)	
H3	0.4799	0.1099	0.4509	0.067*	
C4	0.5814 (3)	0.0464 (4)	0.3743 (2)	0.0560 (9)	
C5	0.6608 (3)	0.0938 (4)	0.3271 (2)	0.0594 (10)	
H5	0.6920	0.0341	0.2953	0.071*	
C6	0.6943 (3)	0.2273 (4)	0.32633 (18)	0.0503 (8)	
H6	0.7479	0.2566	0.2944	0.060*	
C7	0.5484 (4)	−0.1011 (4)	0.3759 (3)	0.0888 (14)	
H7A	0.5897	−0.1488	0.3410	0.133*	
H7B	0.4596	−0.1117	0.3637	0.133*	
H7C	0.5750	−0.1364	0.4241	0.133*	
C8	1.4014 (3)	1.0844 (3)	0.16123 (17)	0.0428 (8)	
H8	1.4868	1.0709	0.1622	0.051*	
C9	1.3610 (3)	1.1564 (3)	0.21756 (16)	0.0397 (7)	
H9	1.4190	1.1919	0.2548	0.048*	
C10	1.2347 (3)	1.1757 (3)	0.21868 (15)	0.0341 (7)	
C11	1.1536 (3)	1.1223 (3)	0.16073 (16)	0.0367 (7)	
H11	1.0678	1.1325	0.1593	0.044*	
C12	1.1999 (3)	1.0545 (3)	0.10545 (17)	0.0399 (7)	
H12	1.1438	1.0216	0.0665	0.048*	
C13	1.1866 (3)	1.2496 (3)	0.28054 (17)	0.0463 (8)	
H13A	1.1419	1.3292	0.2610	0.056*	
H13B	1.2573	1.2789	0.3155	0.056*	
C14	1.0998 (3)	1.1642 (3)	0.32062 (17)	0.0482 (8)	
H14A	1.0670	1.2188	0.3571	0.058*	
H14B	1.0297	1.1331	0.2858	0.058*	
C15	1.1673 (3)	1.0460 (4)	0.3572 (2)	0.0637 (10)	
H15A	1.2369	1.0792	0.3916	0.076*	
H15B	1.2021	0.9947	0.3201	0.076*	
C16	1.0919 (3)	0.9508 (3)	0.39783 (17)	0.0437 (7)	
C17	0.9734 (3)	0.9066 (3)	0.36888 (18)	0.0476 (8)	
H17	0.9342	0.9404	0.3244	0.057*	
H18	0.8344	0.7824	0.3862	0.057*	
H19	1.1140	0.7734	0.5447	0.057*	
C18	0.9136 (3)	0.8116 (3)	0.40651 (17)	0.0444 (8)	
C19	1.0775 (3)	0.8061 (3)	0.49934 (17)	0.0488 (8)	
C20	1.1415 (3)	0.8990 (3)	0.46477 (18)	0.0496 (8)	
H20	1.2198	0.9276	0.4869	0.060*	
N1	1.3229 (2)	1.0335 (2)	0.10532 (13)	0.0381 (6)	
N2	0.9650 (2)	0.7598 (2)	0.47099 (13)	0.0405 (6)	
O1	0.6460 (2)	0.5548 (2)	0.43292 (12)	0.0563 (6)	
O2	0.6561 (2)	0.5423 (3)	0.30278 (13)	0.0676 (7)	
O3	0.8348 (2)	0.4792 (2)	0.39088 (14)	0.0605 (6)	
S1	0.69972 (7)	0.48821 (8)	0.37467 (4)	0.0449 (2)	
Ag1	0.88538 (2)	0.59100 (3)	0.521998 (14)	0.05157 (12)	

### Atomic displacement parameters (Å^2^)

**Table:**

	*U*^11^	*U*^22^	*U*^33^	*U*^12^	*U*^13^	*U*^23^
C1	0.0369 (16)	0.0487 (19)	0.0340 (16)	−0.0031 (14)	−0.0054 (13)	0.0006 (15)
C2	0.0455 (18)	0.0487 (19)	0.0433 (19)	−0.0037 (15)	0.0037 (15)	−0.0019 (16)
C3	0.050 (2)	0.059 (2)	0.057 (2)	−0.0110 (17)	0.0033 (17)	0.0091 (19)
C4	0.050 (2)	0.053 (2)	0.060 (2)	−0.0068 (17)	−0.0107 (18)	−0.0034 (19)
C5	0.054 (2)	0.062 (2)	0.058 (2)	0.0060 (18)	−0.0060 (18)	−0.0189 (19)
C6	0.0407 (18)	0.063 (2)	0.0458 (19)	−0.0025 (16)	0.0026 (15)	−0.0042 (17)
C7	0.097 (3)	0.056 (3)	0.110 (4)	−0.018 (2)	0.003 (3)	−0.011 (2)
C8	0.0400 (17)	0.0460 (18)	0.0442 (19)	0.0039 (14)	0.0117 (15)	0.0086 (15)
C9	0.0445 (18)	0.0393 (17)	0.0344 (16)	−0.0052 (14)	0.0030 (14)	0.0018 (14)
C10	0.0501 (18)	0.0226 (14)	0.0316 (15)	−0.0035 (13)	0.0127 (13)	0.0041 (12)
C11	0.0380 (16)	0.0343 (16)	0.0388 (17)	0.0026 (13)	0.0089 (14)	0.0025 (13)
C12	0.0430 (18)	0.0404 (17)	0.0356 (17)	−0.0005 (14)	0.0039 (13)	−0.0004 (14)
C13	0.065 (2)	0.0344 (17)	0.0440 (18)	−0.0016 (15)	0.0219 (16)	−0.0040 (15)
C14	0.065 (2)	0.0439 (19)	0.0401 (18)	−0.0010 (16)	0.0228 (16)	−0.0050 (15)
C15	0.053 (2)	0.074 (3)	0.066 (2)	−0.0001 (19)	0.0129 (18)	0.026 (2)
C16	0.0435 (18)	0.0434 (18)	0.0454 (19)	−0.0012 (15)	0.0108 (15)	0.0054 (15)
C17	0.052 (2)	0.050 (2)	0.0389 (18)	0.0011 (16)	0.0008 (15)	0.0124 (15)
C18	0.0397 (17)	0.0474 (19)	0.0441 (19)	−0.0037 (15)	−0.0006 (14)	0.0012 (16)
C19	0.057 (2)	0.053 (2)	0.0336 (17)	−0.0044 (17)	−0.0048 (15)	0.0093 (16)
C20	0.0428 (18)	0.056 (2)	0.047 (2)	−0.0088 (16)	−0.0048 (15)	0.0068 (16)
N1	0.0487 (15)	0.0344 (13)	0.0325 (14)	0.0042 (12)	0.0108 (12)	0.0028 (11)
N2	0.0493 (15)	0.0390 (14)	0.0334 (14)	−0.0046 (12)	0.0064 (12)	0.0024 (12)
O1	0.0720 (16)	0.0508 (14)	0.0460 (14)	−0.0014 (12)	0.0079 (12)	−0.0029 (11)
O2	0.0875 (18)	0.0722 (17)	0.0398 (14)	−0.0038 (14)	−0.0025 (12)	0.0167 (13)
O3	0.0428 (13)	0.0628 (15)	0.0735 (17)	−0.0153 (11)	0.0002 (12)	0.0014 (14)
S1	0.0475 (5)	0.0484 (5)	0.0371 (4)	−0.0078 (4)	0.0002 (3)	0.0058 (4)
Ag1	0.0679 (2)	0.04608 (18)	0.04440 (18)	−0.00999 (12)	0.02079 (13)	0.00570 (12)

### Geometric parameters (Å, º)

**Table:**

C1—C2	1.388 (4)	C13—H13A	0.9700
C1—C6	1.390 (4)	C13—H13B	0.9700
C1—S1	1.775 (3)	C14—C15	1.493 (5)
C2—C3	1.379 (5)	C14—H14A	0.9700
C2—H2	0.9300	C14—H14B	0.9700
C3—C4	1.385 (5)	C15—C16	1.515 (4)
C3—H3	0.9300	C15—H15A	0.9700
C4—C5	1.389 (5)	C15—H15B	0.9700
C4—C7	1.511 (5)	C16—C20	1.373 (4)
C5—C6	1.377 (5)	C16—C17	1.387 (4)
C5—H5	0.9300	C17—C18	1.385 (4)
C6—H6	0.9300	C17—H17	0.9300
C7—H7A	0.9600	C18—N2	1.341 (4)
C7—H7B	0.9600	C18—H18	0.9295
C7—H7C	0.9600	C19—N2	1.334 (4)
C8—N1	1.338 (4)	C19—C20	1.365 (4)
C8—C9	1.382 (4)	C19—H19	0.9301
C8—H8	0.9300	C20—H20	0.9300
C9—C10	1.381 (4)	N1—Ag1^i^	2.155 (2)
C9—H9	0.9300	N2—Ag1	2.162 (2)
C10—C11	1.386 (4)	O1—S1	1.451 (2)
C10—C13	1.510 (4)	O2—S1	1.445 (2)
C11—C12	1.374 (4)	O3—S1	1.451 (2)
C11—H11	0.9300	O3—Ag1	2.645 (2)
C12—N1	1.346 (4)	Ag1—N1^ii^	2.155 (2)
C12—H12	0.9300	Ag1—Ag1^iii^	3.2628 (5)
C13—C14	1.532 (4)		
			
C2—C1—C6	118.8 (3)	C15—C14—C13	111.2 (3)
C2—C1—S1	121.6 (2)	C15—C14—H14A	109.4
C6—C1—S1	119.5 (2)	C13—C14—H14A	109.4
C3—C2—C1	120.0 (3)	C15—C14—H14B	109.4
C3—C2—H2	120.0	C13—C14—H14B	109.4
C1—C2—H2	120.0	H14A—C14—H14B	108.0
C2—C3—C4	121.9 (3)	C14—C15—C16	116.9 (3)
C2—C3—H3	119.1	C14—C15—H15A	108.1
C4—C3—H3	119.1	C16—C15—H15A	108.1
C3—C4—C5	117.5 (3)	C14—C15—H15B	108.1
C3—C4—C7	121.8 (4)	C16—C15—H15B	108.1
C5—C4—C7	120.7 (4)	H15A—C15—H15B	107.3
C6—C5—C4	121.4 (3)	C20—C16—C17	116.5 (3)
C6—C5—H5	119.3	C20—C16—C15	120.7 (3)
C4—C5—H5	119.3	C17—C16—C15	122.7 (3)
C5—C6—C1	120.4 (3)	C18—C17—C16	119.7 (3)
C5—C6—H6	119.8	C18—C17—H17	120.2
C1—C6—H6	119.8	C16—C17—H17	120.2
C4—C7—H7A	109.5	N2—C18—C17	122.8 (3)
C4—C7—H7B	109.5	N2—C18—H18	118.5
H7A—C7—H7B	109.5	C17—C18—H18	118.7
C4—C7—H7C	109.5	N2—C19—C20	123.0 (3)
H7A—C7—H7C	109.5	N2—C19—H19	118.5
H7B—C7—H7C	109.5	C20—C19—H19	118.5
N1—C8—C9	122.7 (3)	C19—C20—C16	121.0 (3)
N1—C8—H8	118.6	C19—C20—H20	119.5
C9—C8—H8	118.6	C16—C20—H20	119.5
C10—C9—C8	120.1 (3)	C8—N1—C12	117.3 (3)
C10—C9—H9	119.9	C8—N1—Ag1^i^	122.5 (2)
C8—C9—H9	119.9	C12—N1—Ag1^i^	120.1 (2)
C9—C10—C11	116.9 (3)	C19—N2—C18	117.0 (3)
C9—C10—C13	121.8 (3)	C19—N2—Ag1	119.6 (2)
C11—C10—C13	121.3 (3)	C18—N2—Ag1	122.9 (2)
C12—C11—C10	120.1 (3)	O2—S1—O3	113.49 (15)
C12—C11—H11	119.9	O2—S1—O1	113.29 (15)
C10—C11—H11	119.9	O3—S1—O1	111.93 (15)
N1—C12—C11	122.8 (3)	O2—S1—C1	106.18 (14)
N1—C12—H12	118.6	O3—S1—C1	104.30 (14)
C11—C12—H12	118.6	O1—S1—C1	106.81 (14)
C10—C13—C14	113.2 (2)	N1^ii^—Ag1—N2	160.18 (9)
C10—C13—H13A	108.9	N1^ii^—Ag1—Ag1^iii^	100.65 (7)
C14—C13—H13A	108.9	N2—Ag1—Ag1^iii^	87.67 (6)
C10—C13—H13B	108.9	N1^ii^—Ag1—O3	111.38 (8)
C14—C13—H13B	108.9	N2—Ag1—O3	88.42 (8)
H13A—C13—H13B	107.7		

Symmetry codes: (i) *x*+1/2, −*y*+3/2, *z*−1/2; (ii) *x*−1/2, −*y*+3/2, *z*+1/2; (iii) −*x*+2, −*y*+1, −*z*+1.

### Hydrogen-bond geometry (Å, º)

**Table:**

*D*—H···*A*	*D*—H	H···*A*	*D*···*A*	*D*—H···*A*
C14—H14*B*···O2^iv^	0.97	2.56	3.533 (4)	178
C12—H12···O1^i^	0.93	2.58	3.332 (4)	138
C8—H8···O3^v^	0.93	2.46	3.305 (4)	151

Symmetry codes: (i) *x*+1/2, −*y*+3/2, *z*−1/2; (iv) −*x*+3/2, *y*+1/2, −*z*+1/2; (v) −*x*+5/2, *y*+1/2, −*z*+1/2.
